# Image Enhancement via Special Functions and Its Application for Near Infrared Imaging

**DOI:** 10.1002/gch2.202200179

**Published:** 2023-05-24

**Authors:** Ruoxi Yang, Long Chen, Ling Zhang, Zongan Li, Yingcheng Lin, Ye Wu

**Affiliations:** ^1^ School of Electrical and Automation Engineering Nanjing Normal University Nanjing 210046 China; ^2^ College of Microelectronics and Communication Engineering Chongqing University Chongqing 400044 China

**Keywords:** filters, image enhancement, near infrared imaging, noise suppression, special function

## Abstract

Image enhancement is important given that it can be used to highlight the area of interest in the images. This article designs four filters via special function for realizing image enhancement. Firstly, a filter based on the exponential function is designed. When the value of the progression is even, the edge feature can be extracted. When the value of the progression is odd, sharp contrast can be obtained. Secondly, a filter is built using hyperbolic cosine and its inverse function, where a printmaking feature can be extracted. Thirdly, a filter is made via a hyperbolic secant function and its inverse. It can lead to the extraction of image edge. When the progression value is increasing, marginal effect can be found and the brightness is decreasing. Ripple morphology can be found. Fourthly, a filter is constructed through a hyperbolic sine function and its inverse, where marginal features can be extracted. Furthermore, these filters are useful for extracting the marginal features even when a high noise density of 0.9 is added to the original images. They are useful for highlighting the images acquired from near infrared imaging.

## Graphical abstract

Image enhancement is crucial given that it can considerably lower the amount of information that the computer will process and highlight the area of interest in the images. This article reports the design of four filters via special function for realizing image enhancement. They are shown to be useful for highlighting the images acquired from near infrared imaging.

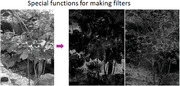

## 1 Introduction

An image can be considered as a 2D signal system, whose processing is considered globally and technically critical. Image enhancement is an important foundation in the field of image analysis such as image segmentation, target area recognition, and area shape extraction. It greatly reduces the amount of information that the computer has to process and can meet the high set of image features required by machine vision. This is useful in the current age of internet of things, where the interplay between the image and human is huge.

Many groups have been active in this research subject. For instance, Z. Lu et al. used the method of quantum computation to extract image edge. Basically, they designed an algorithm of flexible representation of quantum.^[^ ^1^ ^]^ They simply use quantum flexibility to represent the image, whose pixels are processed as quantum states sequence. This provides amazing computational efficiency. A. Bozorgmehr et al. made a digital fuzzy model for getting the information of the image edge, which is implemented via using carbon nanotube field effect transistor.^[^ ^2^ ^]^ It has shown the merit of low power consumption and good accuracy.

The technique of neural network is wildly used for image enhancement.^[^ ^3^, ^4^, ^5^, ^6^, ^7^, ^8^ ^]^ For example, B. Wang et al. built a spiking neuron for finding the edge of infrared images.^[^ ^3^ ^]^ Z.‐F. Wang et al. used convolutional neural network to exact the defect in a thermal image.^[^ ^4^ ^]^

Profile extraction from the near‐infrared image has been a research topic of long‐term interest.^[^ ^9^ ^]^ Infrared images hold rough characteristics, which usually contain large volume of noise and features in low contrast. This is especially harmful in medical applications. When doctors need to grade the pathological tissues using the microscopic images, they find that unclear texture and blurred edges are presented in the images, which are destructive for acquiring information. They find that it is hard to get the diagnosis results. Therefore, image enhancement is required for reducing noise, optimizing image quality, and getting core morphology. The enhancement of infrared image faces several challenges. Generally, people find that it is difficult to increase the sharpness of the image edges, to stretch the gray‐scale, and to smash the noise. Especially, it has been causing tremendous hardships for getting rid of noise and highlighting edge in these images because the noise and the edge are in the same region of high frequency.^[^ ^9^ ^]^

Traditional ways of profile extraction include the differential operators of Roberts, Prewitt, Sobel, Canny, Laplacian, and LoG. They are reported to show several drawbacks^[^ ^6^ ^]^:

- The use of Roberts operator can lead to rough edge and incorrect edge regions.
- The use of Prewitt operator can result in very wide range and interrupted features.
- The use of Sobel operator can present wrong regions of edge.
- The Laplacian operator can bring the corrupted edge profile related with the noise.
- The LoG operator cannot be used to get rid of the salt‐and‐pepper noise.
- The Canny operator cannot have good results if there is a big difference on the gray‐level variations between the background and the subjects.

Since the use of the traditional operators cannot have accurate profile extraction, researchers have come up with various novel ways for the image enhancement.^[^ ^13^, ^14^, ^15^, ^16^, ^17^, ^18^, ^19^, ^20^, ^21^, ^22^, ^23^, ^24^, ^25^, ^26^, ^27^, ^28^, ^29^, ^30^, ^31^ ^]^ Among them, mathematical method has been intensively used for image enhancement. One good example is shown in the application of the Hausdorff derivative, which is used for image edge extraction.^[^ ^10^ ^]^ It has been proven that Hausdorff derivative gradient method is better than the Sobel/Canny approach. It is interesting to find out that nonlinear diffusion equations are useful for smoothening and sharpening the edge of the image.^[^ ^11^ ^]^ Another example can be found in the use of cooperative game formulation, which is shown to be effective in image edge detection.^[^ ^12^ ^]^

The other research efforts for acquiring the image enhancement involved with the method of using wavelets.^[^ ^13^, ^14^, ^15^, ^16^, ^17^, ^18^, ^19^, ^20^, ^21^, ^22^, ^23^, ^24^, ^25^, ^26^, ^27^, ^28^, ^29^, ^30^, ^31^ ^]^ The wavelets are generally wave‐like oscillatory functions. They show a recovery impact since the features acquired via the wavelets show different resolutions. Their multi‐scale components are constructed in very short duration, which makes them very ideal for processing the noisy images. They can be used to highlight the subjects of interest in the figures. Moreover, the gray levels required can be stretched and the uninteresting gray levels can be reduced.^[^ ^13^, ^14^, ^15^ ^]^ In terms of their operation, the wavelets are associated with multi‐scale functions. They are used via the combination of spatial filtering in order to find out the difference in various scales. They used a technique of tracking the edge of a high‐frequency range of frequency domain. Basically, they first divide an image into different bands, then each band is cut into various windows. Finally, each window is calculated via the functions. This way of operation can be used for processing the subjects holding interrupted features.^[^ ^16^, ^17^, ^18^, ^19^ ^]^ Especially, it is reported that the traditional wavelets can be modified to contain the directional factors,^[^ ^15^ ^]^ which can have direction selectivity. These functional modifications can lead to clear and continuous margin.

This inspires us that special functions may possibly provide good way of image enhancement. We hypothesized the development of special‐functions‐based filters that would (1) solve the edge detection problems associated with the noisy figures and (2) allow for the customization of activated morphological probes for reliable edge profiling. To accomplish this, we herein report four types of special function filters to perform enhancement on images.

It can be expected that pixel values from the images can be transformed into the special functions. Therefore, either the modification of the pixel values or its frequency counterparts from the Fourier transform can bring new filters. The basic theory of the near‐infrared‐image denoising can be mathematically considered as solving the problem: *I = ФI_0_+Х*, where *Ф* is a denoising matrix, *I*_0_ is original image, and *I* is the noise‐corrupted image. In our testing of various functions, we have obtained four special functions that are capable of getting rid of the noise and enhancing the image true feature. In this work, we achieve an innovative framework for extracting important or marginal profiles from the near infrared image. Our work provides a simple platform whose parameters can be changed in order to enhance the images with a high level of Gaussian noise.

It should be mentioned that our method is supplementary to current approaches for the image enhancement. They show flexible mathematics that can be adjustable for the purpose of processing different images. The building of these functions will not destroy the foundation of the image enhancement. Simply, these new weapons are adding to the arsenal of the image enhancement.

The next section is about the modeling of four filters, which are built by four special functions. In Section 3, a set of random images are chosen to be processed by these four filters. Feature of the images can be extracted effectively and these enable image enhancement. In Section 4, their effective application in near infrared imaging is shown. Finally, we shall discuss the merits of using these filters for processing the images. Indeed, these may be possibly useful in a very wide range of applications, including artificial intelligence, automatic driving, target recognition, mechanical vision, medical, and other fields.

## 2 Experimental Section

A framework for image enhancement was worked out. It is often difficult to produce clear edge and correct profile in a large and complex image. A new operator was presented that can extract the profile in very narrow range. Moreover, a framework was developed that could process the image when extensive noise was presented. Furthermore, the frameworks that can be mathematically modified in a flexible way were considered so that they can be adapted to different images. They showed flexible mathematics that can be adjustable for the purpose of processing different images.

### 2.1 An “Exp” Filter

An exponential filter was proposed, which can detect image edge. The special function used is as follows:

(1) $$\begin{array}{c} u\left(m,n\right)=a\times e^{-\frac{q{\left(tt(m,n)-b\right)}^{n_{2}}}{c^{2}}} \end{array}$$

Here, *tt(m,n)* is pixels value before filtering, *u(m,n)* is pixels value after filtering; *q*, *b*, *n_2_* and *c* are constants. It can be seen that its form is similar to the Gaussian function.

It should be noted that the image was first converted to a gray‐scale image in order to reduce the amount of data computed by the exponential function filter. In addition, in order to prevent the image data from exceeding the range and being detrimental to subsequent operations, the image data was converted to double precision for subsequent filtering. The detailed algorithm is shown in Algorithm 1.

Algorithm 1 **The algorithm of the “exp” filter for the image enhancement**

**Table:** 

1:	Read the image in JPG format
2:	Converting the image into a gray image
3:	Defining the parameters h and w representing size of the image
4:	**for** each iteration m = 3 to h‐1 do
5:	**for** each iteration n = 3 to w‐1 do
6:	Calculating the new pixel value: $u\left(m,n\right)=a\times e^{-\frac{q{\left(tt(m,n)-b\right)}^{n_{2}}}{c^{2}}}$_._
7:	**end for**
8:	**end for**
9:	Showing the processed image

### 2.2 An “Cosh‐Acosh” Filter

Hyperbolic functions such as hyperbolic cosine and its inverse function were used to construct a “cosh‐acosh” filter.

First *D* is defned as,

(2) $$\begin{array}{c} D=\sqrt{{\left(i-m_{1}\right)}^{n3}+{\left(j-n_{1}\right)}^{n3}} \end{array}$$

Here, *i* is a horizontal axis pixel, *j* is the vertical axis pixel, *m_1_* is one‐half of the total pixels in the horizontal axis of the image matrix, *n_1_* is one‐half of the total pixels in the vertical axis pixel. *n3* is a constant.

Then, *CNx* is defined as,

(3) $$\begin{array}{c} CNx=\cosh\left(n_{1}\times a\cosh\left({\left(\frac{D_{0}}{D}\right)}^{n5}\right)\right) \end{array}$$

Here, *D_0_* is 0.05 pixels in the horizontal axis of the image matrix.

Moreover, *h3* is defined as,

(4) $$\begin{array}{c} h3=\frac{1}{1+\varepsilon^{2}\times CNx^{n4}} \end{array}$$

Fourier transformation was applied to the original figure, which gave a set of 2D frequencies value *g2(i,j)*. *h3* in Equation (4) was applied to *g2(i,j)* that resulted in another set of frequencies value *s3(i,j)*. This means that

(5) $$\begin{array}{c} s3\;\left(i,j\right)=h3\times g2\left(i,j\right) \end{array}$$

Then, the inverse transformation of *s3(i,j)* led to a filtered image. The corresponding algorithm is shown in Algorithm 2.

Algorithm 2 **The algorithm of the “cosh‐acosh” filter for the image enhancement**

**Table:** 

1:	Read the image in JPG format
2:	Converting the image into a gray image
3:	Using Fourier transform to convert the image into the 2D frequency range
4:	Defining the parameters M and N representing size of the frequency range
5:	**for** each iteration i = 1 to M do
5:	**for** each iteration j = 1 to N do
6:	Defining all the constants, calculating the function *h3*: $h3=\frac{1}{1+\varepsilon^{2}\times CNx^{n4}}$_._
7:	Obtaining the processed frequency values *s3(i,j)*: s3(i,j) = h3*g2(i,j).
8:	**end for**
9:	**end for**
10:	Using inverse Fourier transform to obtain the new image‐pixels value
11:	Showing the processed image

### 2.3 An “Sech‐Asech” Filter

The construction of a “sech‐asech” filter was similar to that of the “cosh‐acosh” filter. Hyperbolic functions *CNx_2_* is defined as,

(6) $$\begin{array}{c} CNx_{2}={\left(\mathrm{sech}\left(\lambda \times a\mathrm{sech}\left(\frac{D_{0}}{D}\right)\right)\right)}^{nn} \end{array}$$

*h4* is another function related with *CNx_2_*:

(7) $$\begin{array}{c} h4=\frac{1}{1+\varepsilon^{2}\times CNx_{2}^{2}} \end{array}$$

Here, *λ* and *ε* are constants.

Fourier transformation was used to process the original figure, which generated a set of 2D frequencies value *g2(i,j)*. *h4* in Equation (7) was applied to *g2(i,j)* that generated another set of frequencies value *s4(i,j)*. This can be considered as,

(8) $$\begin{array}{c} s4\;\left(i.j\right)=h4\times g2\left(i,j\right) \end{array}$$

The inverse transformation of *s4(i,j)* can have a filtered image. The detailed algorithm can be found in Algorithm 3.

Algorithm 3 **The algorithm of the “sech‐asech” filter for the image enhancement**

**Table:** 

1:	Read the image in JPG format
2:	Converting the image into a gray image
3:	Using Fourier transform to convert the image into the two‐dimentional frequency range
4:	Defining the parameters M and N representing size of the frequency range
5:	**for** each iteration i = 1 to M do
5:	**for** each iteration j = 1 to N do
6:	Defining all the constants, calculating the function *h4*: $h4=\frac{1}{1+\varepsilon^{2}\times CNx_{2}^{2}}$_._
7:	Obtaining the frequency values *s4(i,j)*: s4(i,j) = h4*g2(i,j).
8:	**end for**
9:	**end for**
10:	Using inverse Fourier transform to get the processed pixels value
11:	Showing the processed image

### 2.4 An “Sinh‐Asinh‐r” Filter

A new pixel value *r (m,n)* is defined as,

(9) $$\begin{array}{c} r\left(m,n\right)=tt{\left(m,n\right)}^{\eta } \end{array}$$

Here, *η* is a constant.

It can be used to generate a new function as in the form of

(10) $$\begin{array}{c} z\left(m,n\right)=\sinh\left(\frac{a_{1}\times a\sinh\left(r\left(m,n\right)+a_{2}\right)}{r{\left(m,n\right)}^{a_{3}}}\right) \end{array}$$

Here, *a_1_*, *a_2_* and *a_3_* are constants. The filtered image can be obtained through *u4 (m,n)*, which can be calculated via

(11) $$\begin{array}{c} u4\left(m,n\right)=z{\left(m,n\right)}^{a_{4}} \end{array}$$

Its algorithm is shown in Algorithm 4.

Algorithm 4 **The algorithm of the “sinh‐asinh‐r” filter for the image enhancement**

**Table:** 

1:	Read the image in JPG format
2:	Converting the image into a gray image
3:	Defining the parameters h and w representing size of the image
5:	**for** each iteration m = 3 to h‐1 do
5:	**for** each iteration n = 3 to w‐1 do
6:	Defining all the constants, calculating the function z(m,n): $z\left(m,n\right)=\sinh\left(\frac{a_{1}\times a\sinh\left(r\left(m,n\right)+a_{2}\right)}{r{\left(m,n\right)}^{a_{3}}}\right)$_._
7:	Obtaining the filtered image u4(m,n): $u4\left(m,n\right)=z{\left(m,n\right)}^{a_{4}}$.
8:	**end for**
9:	**end for**
10:	Showing the processed image

**Figure 1:**
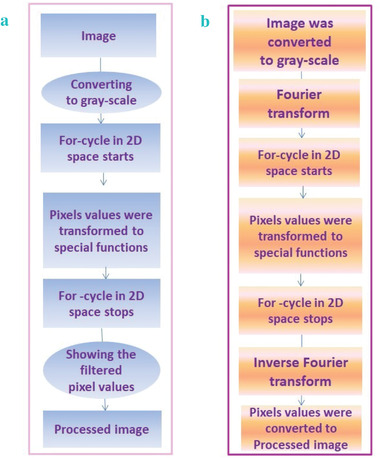
a) The diagram of image processing via the “exp” filter and the “sinh‐asinh‐r” filter. b) The diagram of image processing via the “sech‐asech” filter and the “cosh‐acosh” filter.

Based on the function expression, a special filter was programmed to be used for processing the image. The diagram of processing the image is shown in **Figure** **1**. The special functions were combined with Fourier transform and inverse Fourier transform to get the results when using the “sech‐asech” filter and the “cosh‐acosh” filter.

## 3 Results

### 3.1 Application for Image Enhancement

#### 3.1.1 The “exp” Filter

**Figure 2:**
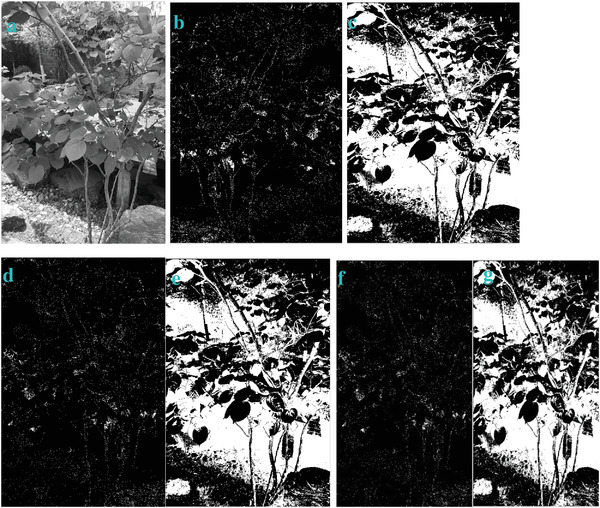
a) Tree image. b) *n_2_* = 2; c) *n_2_* = 7; d) *n_2_* = 8; e) *n_2_* = 9; f) *n_2_* = 28; g) *n_2_* = 29.

**Figure** **2**a is a tree image for processing. Figure 2b–g is the result generated via the “exp” filter when *n_2_* is varying. We found that Figure 2c,e,g shows very sharp contrast between the images and the background compared to other images. They also show clearer, more complete edges within the area. There is a small amount of image information missing in Figure 2b,d,f. As can be seen in the figures, part of the leaf surface is lost.

**Figure 3:**
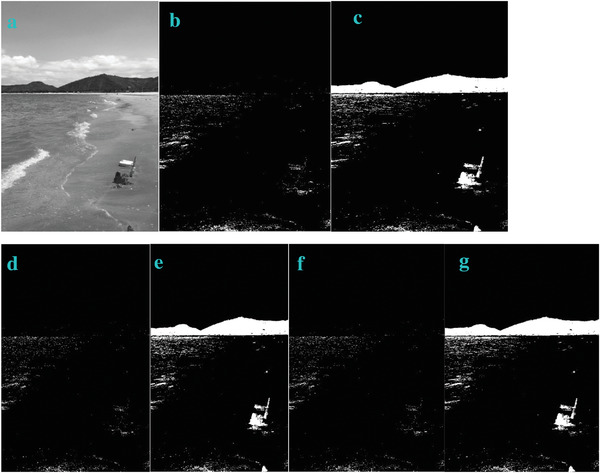
a) Sea image. b) *n_2_* = 2; c) *n_2_* = 7; d) *n_2_* = 8; e) *n_2_* = 9; f) *n_2_* = 28; g) *n_2_* = *29*.

**Figure 4:**
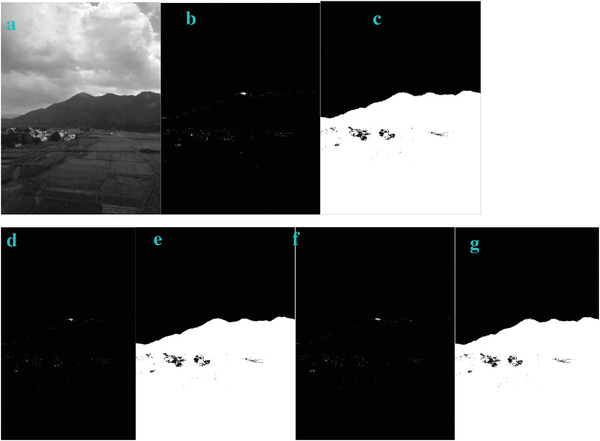
a) Field image. b) *n_2_* = 2; c) *n_2_* = 7; d) *n_2_* = 8; e) *n_2_* = 9; f) *n_2_* = 28; g) *n_2_* = 29.

**Figure 5:**
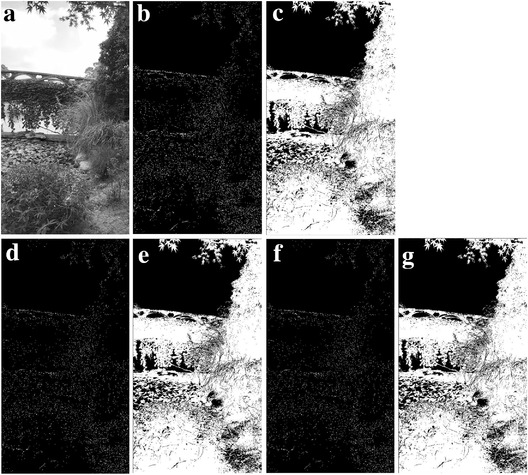
a) Lakeside image. b) *n_2_* = 2; c) *n_2_* = 7; d) *n_2_* = 8; e) *n_2_* = 9; f) *n_2_* = 28; g) *n_2_* = 29.

**Figure 6:**
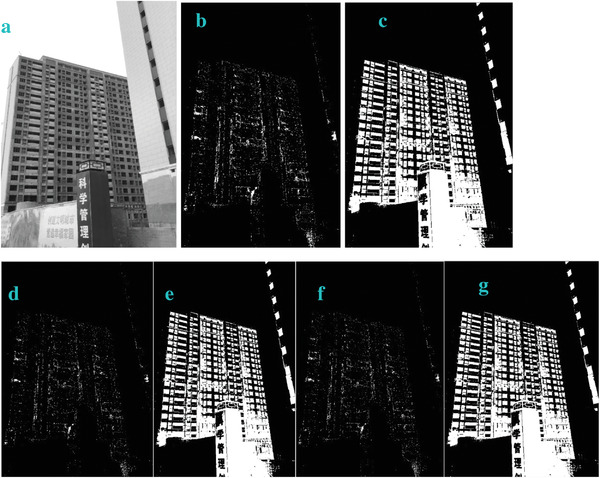
a) Building image. b) *n_2_* = 2; c) *n_2_* = 7; d) *n_2_* = 8; e) *n_2_* = 9; f) *n_2_* = 28; g) *n_2_* = 29.

Another feature we can find is that the brightness of the image is related with *n_2_*. When *n_2_* is even, it is interesting to find out that the image is dark. When *n_2_* is odd, the image is bright. In order to verify this, we also test this trend in other four images of Sea, Field, Lakeside, and Building. They show the similar trend (**Figures** **3**, **4**, **5**, **6**).

**Figure 7:**
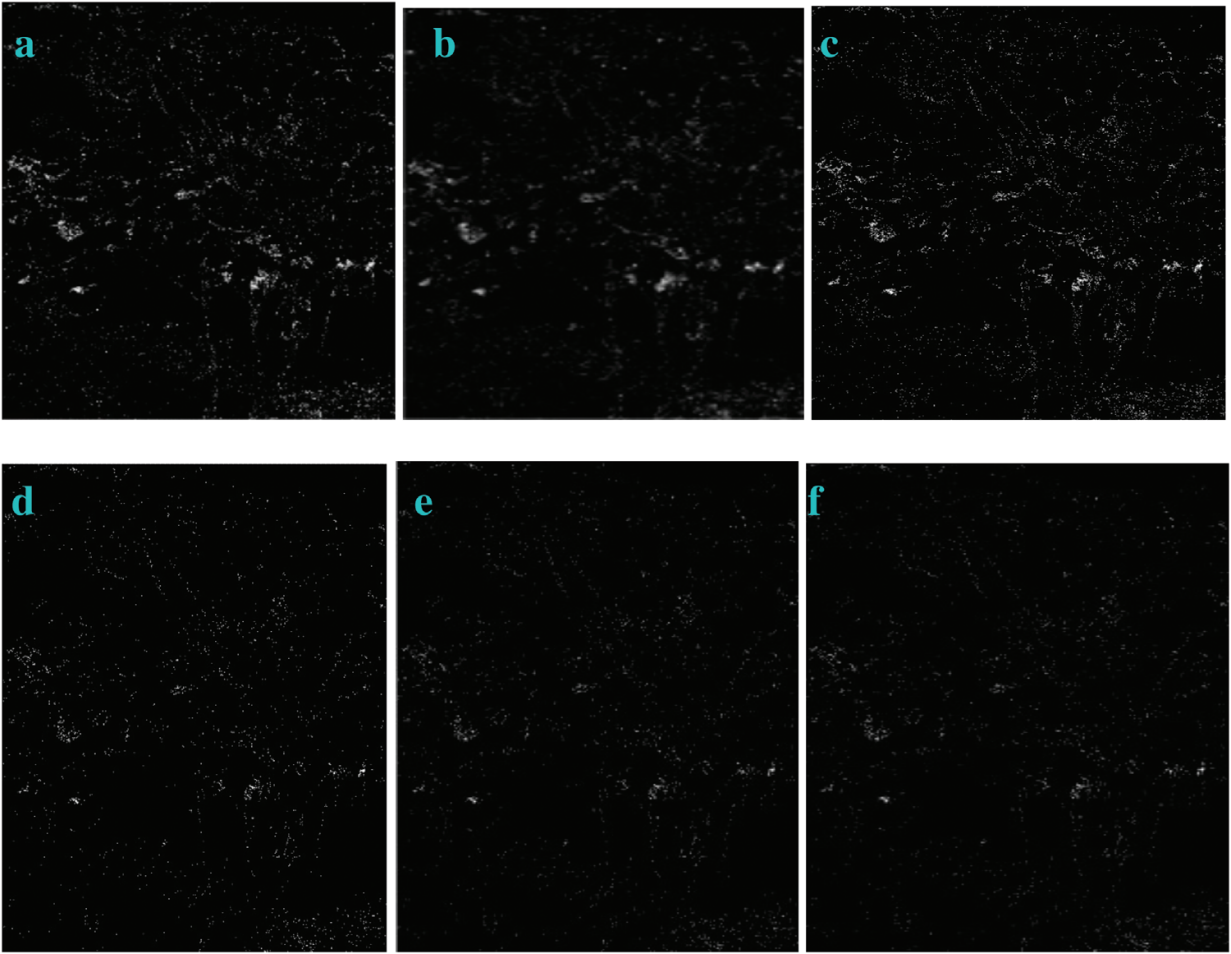
The change of the q value for processing the Tree image: a) *q* = 2; b) *q* = 3; c) *q* = 7; d) *q* = 9; e) *q* = 10; f) *q* = 15.

**Figure 8:**
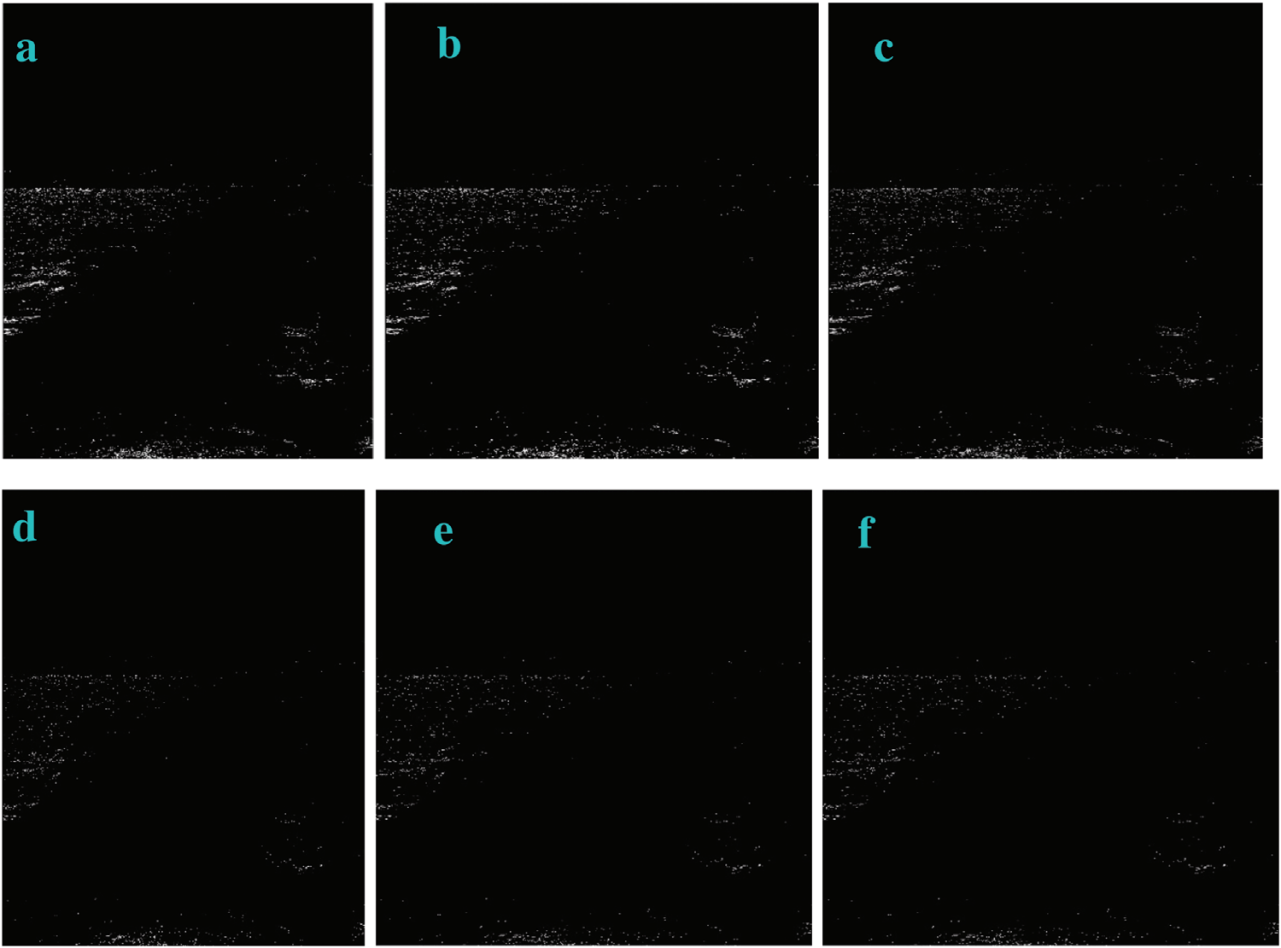
The change of the q value for processing the Sea image: a) *q* = 2; b) *q* = 3; c) *q* = 7; d) *q* = 9; e) *q* = 10; f) *q* = 15.

**Figure 9:**
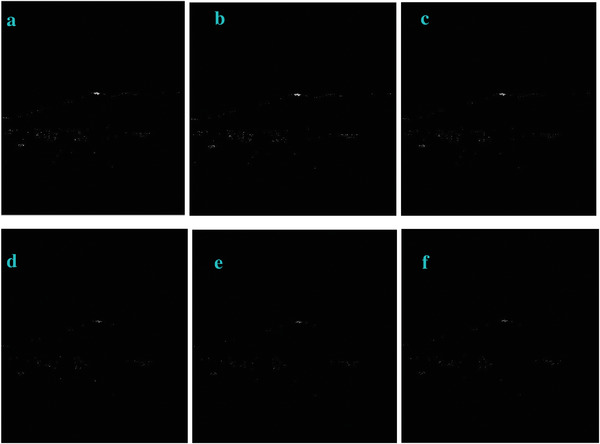
The change of the *q* value for processing the Field image: a) *q* = 2; b) *q* = 3; c) *q* = 7; d) *q* = 9; e) *q* = 10; f) *q* = 15.

**Figure 10:**
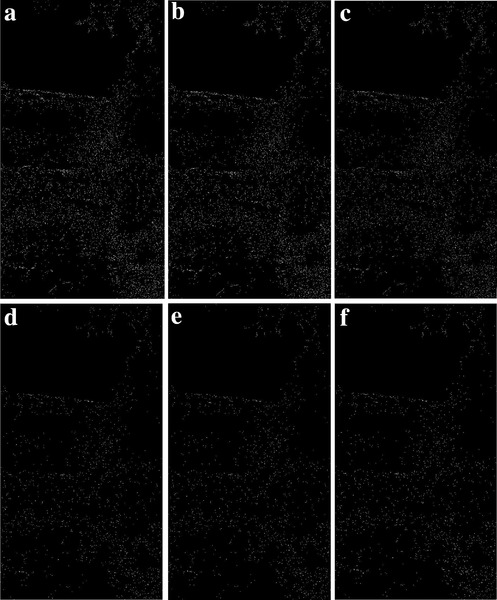
The change of the *q* value for processing the Lakeside image: a) *q* = 2; b) *q* = 3; c) *q* = 7; d) *q* = 9; e) *q* = 10; f) *q* = 15.

**Figure 11:**
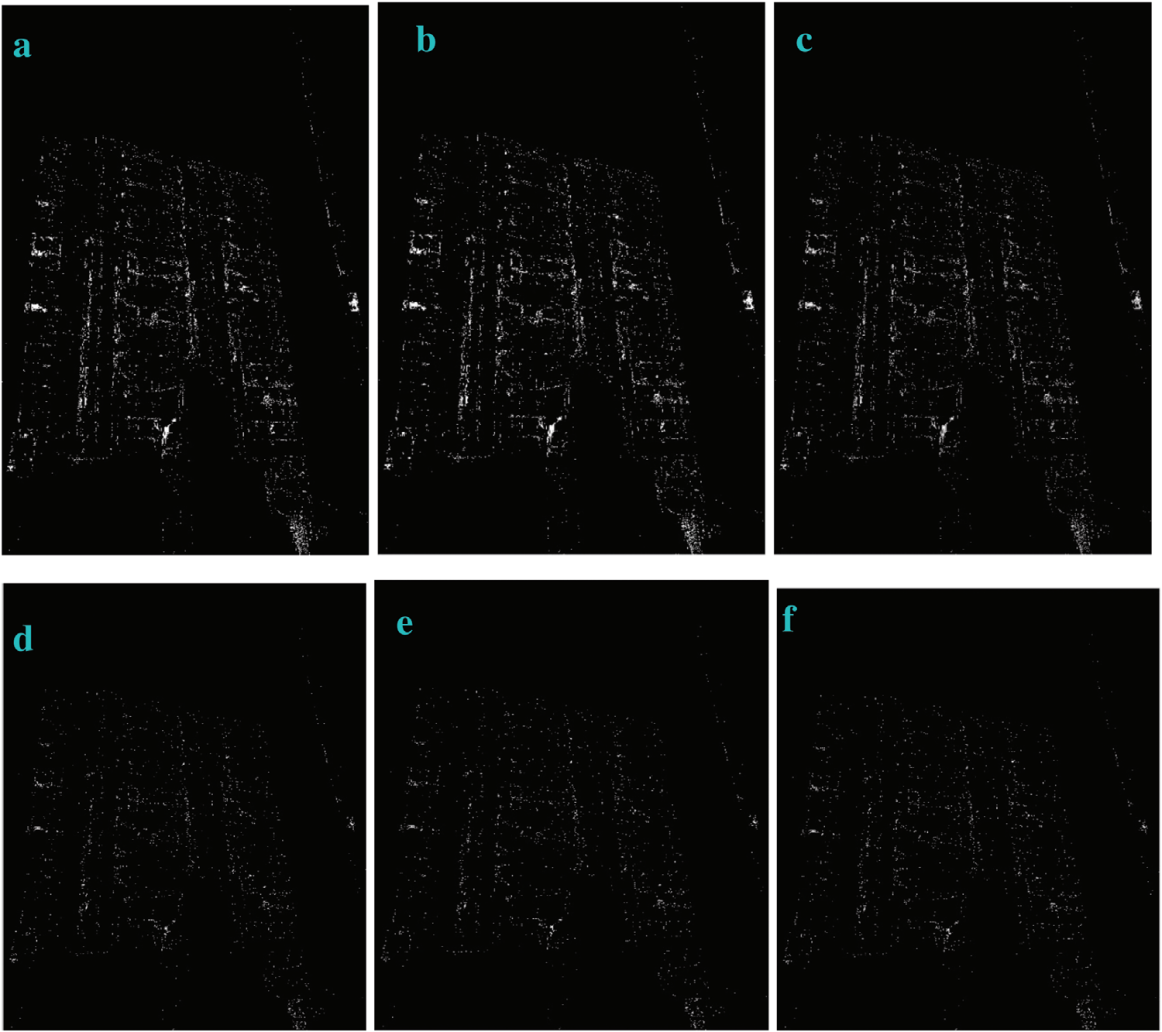
The change of the *q* value for processing the Building image: a) *q* = 2; b) *q* = 3; c) *q* = 7; d) *q* = 9; e) *q* = 10; f) *q* = 15.

We changed the value of *q* in Equation (1) and generated a set of different profile in **Figures** **7**, **8**, **9**, **10**, **11**. It can be seen that as the parameter q increases, the image edge becomes unclear. The greater the value is, the more unclear the image edge is.

**Figure 12:**
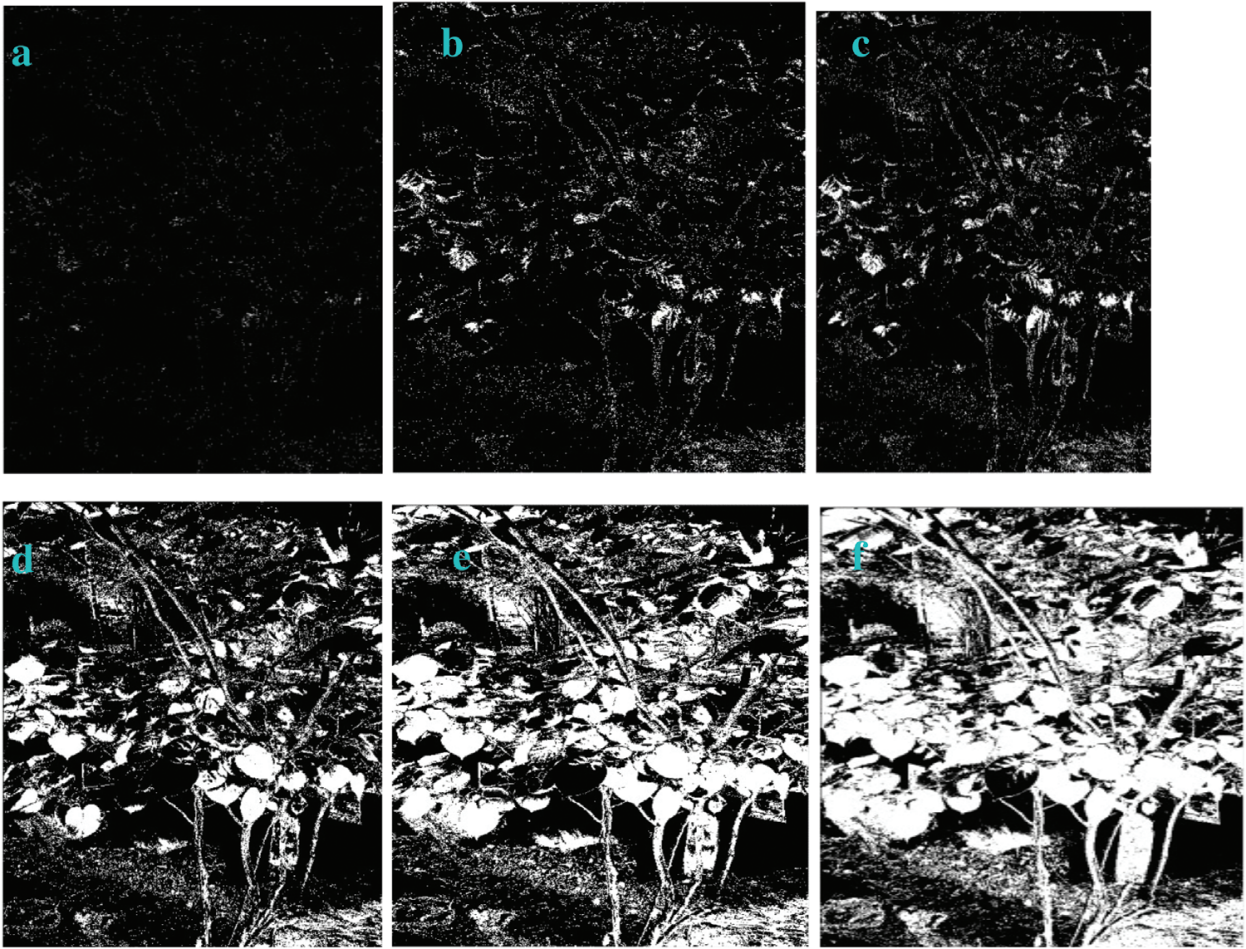
The test of the c value for the Tree image: a) *c* = 0.1; b) *c* = 0.6; c) *c* = 0.9; d) *c* = 3; e) *c* = 5; f) *c* = 7.

**Figure 13:**
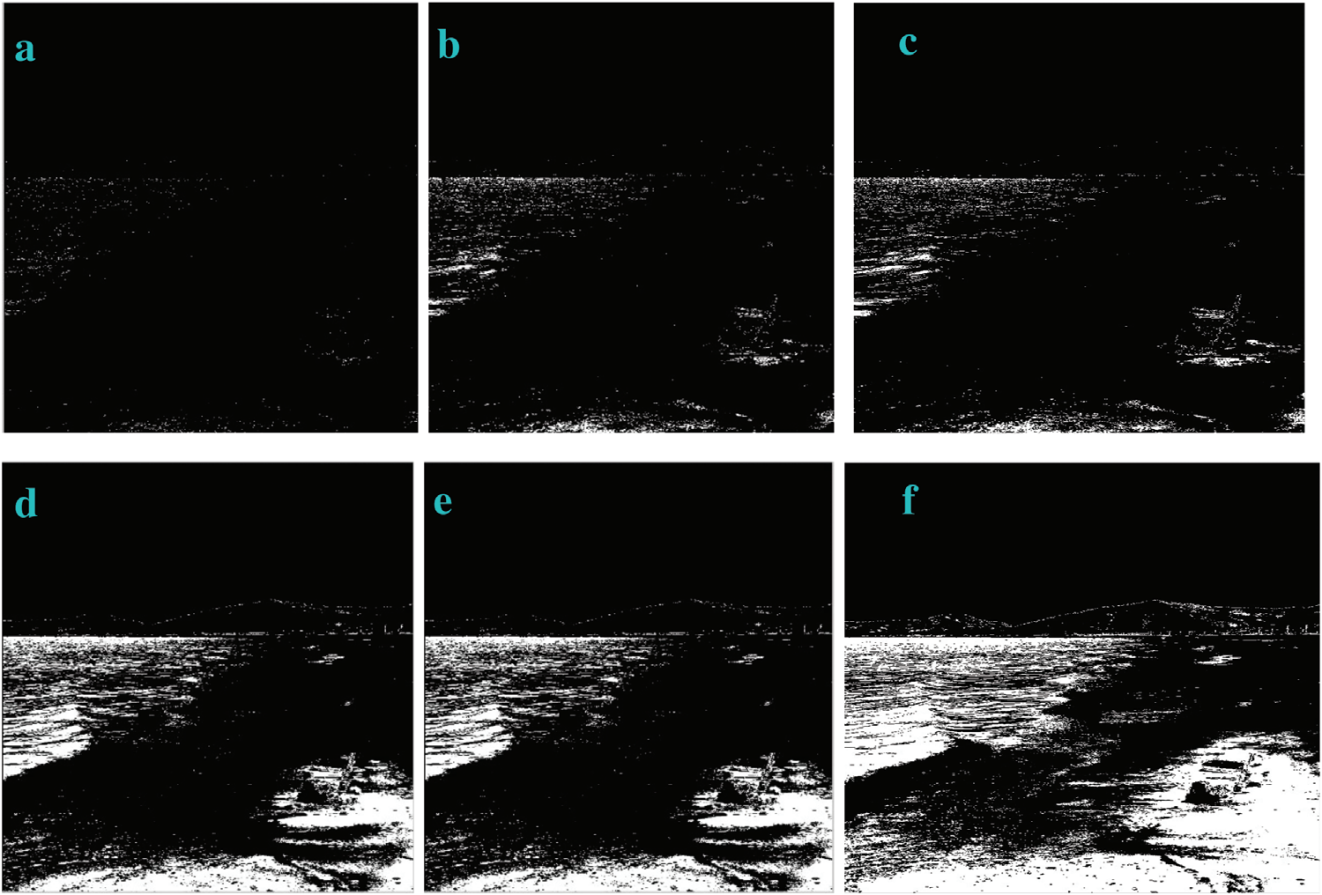
a) *c* = 0.1; b) *c* = 0.6; c) *c* = 0.9; d) *c* = 3; e) *c* = 5; f) *c* = 7.

**Figure 14:**
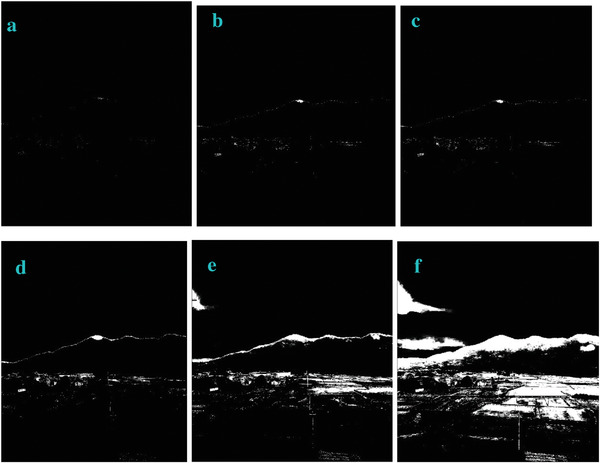
The test of c value for the Field image: a) *c* = 0.1; b) *c* = 0.6; c) *c* = 0.9; d) *c* = 3; e) *c* = 5; f) *c* = 7.

**Figure 15:**
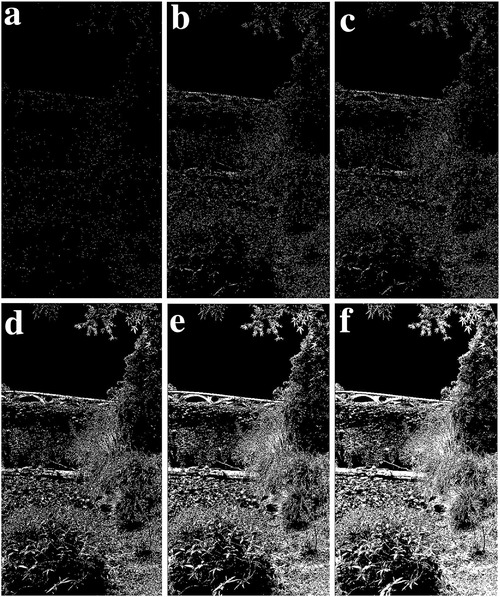
The test of c value for the Lakeside image: a) *c* = 0.1; b) *c* = 0.6; c) *c* = 0.9; d) *c* = 3; e) *c* = 5; f) *c* = 7.

**Figure 16:**
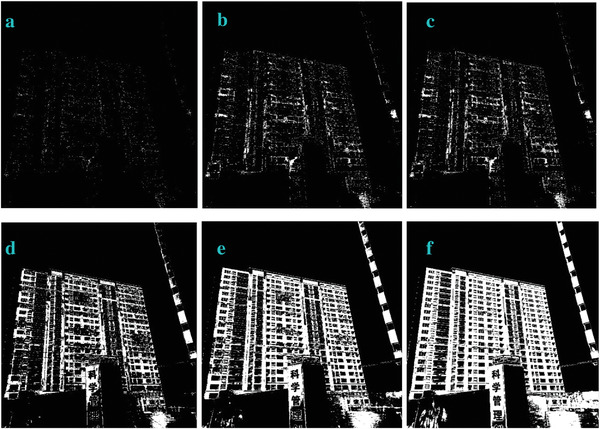
The change of c value for processing the Building image: a) *c* = 0.1; b) *c* = 0.6; c) *c* = 0.9; d) *c* = 3; e) *c* = 5; f) *c* = 7.

As the value of c in Equation (1) is changed, the image filtering effect is shown in **Figures** **12**, **13**, **14**, **15**, **16**. We can find out that as the parameter c increases, the more obvious the contrast between the image and the background, and the more complete the image information extracted is.

#### 3.1.2 The “cosh‐acosh” Filter

**Figure 17:**
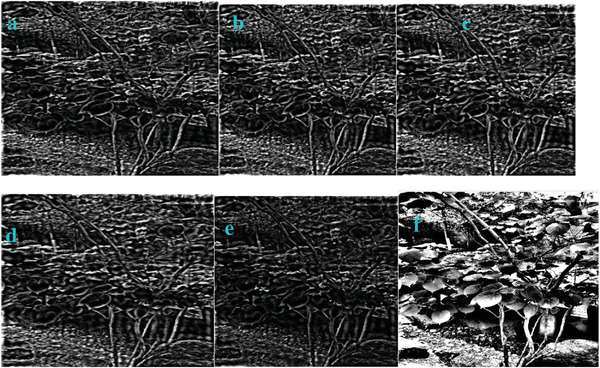
The change of *n5* value for processing the Tree image: a) *n5* = 7; b) *n5* = 17; c) *n5* = 26; d) *n5* = 1000; e) *n5* = 0.1; f) *n5* = 0.0001.

**Figure 18:**
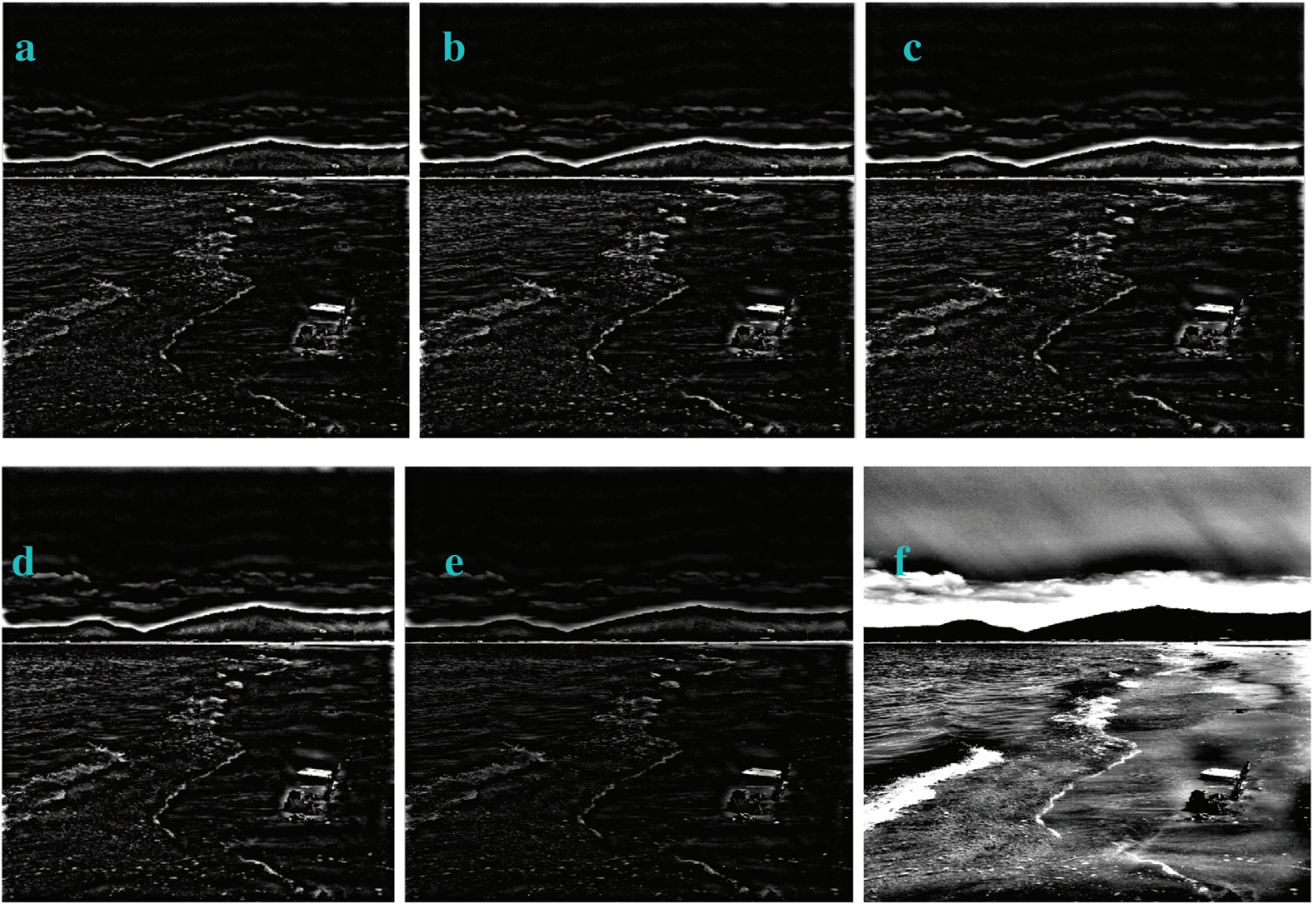
The change of n5 value for processing the Sea image: a) *n5* = 7; b) *n5* = 17; c) *n5* = 26; d) *n5* = 1000; e) *n5* = 0.1; f) *n5* = 0.0001.

**Figure 19:**
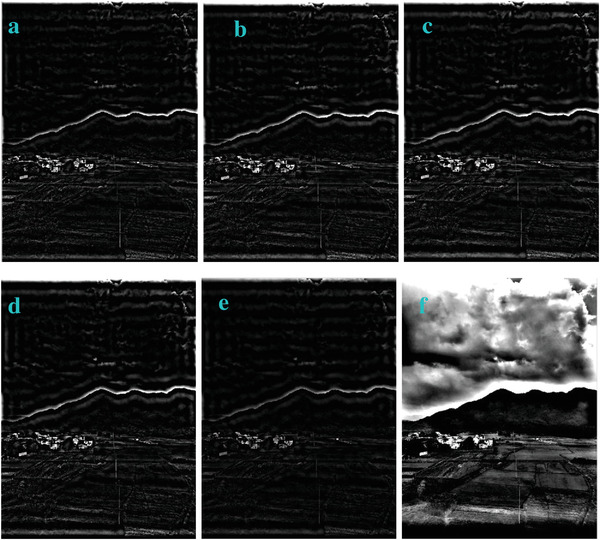
The change of *n5* value for processing the Field image: a) *n5* = 7; b) *n5* = 17; c) *n5* = 26; d) *n5* = 1000; e) *n5* = 0.1; f) *n5* = 0.0001.

**Figure 20:**
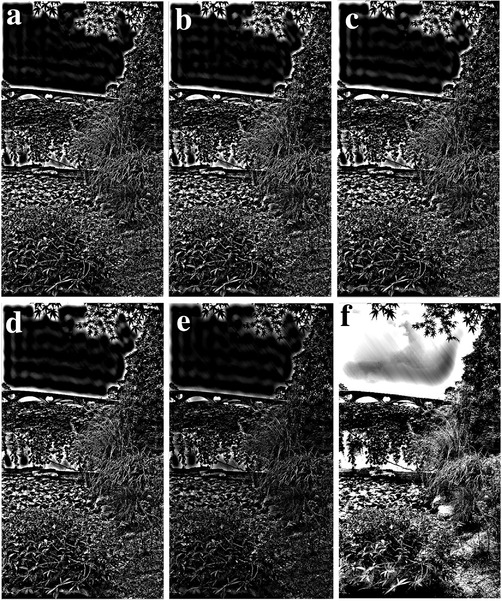
The change of *n5* value for processing the Lakeside image: a) *n5* = 7; b) *n5* = 17; c) *n5* = 26; d) *n5* = 1000; e) *n5* = 0.1; f) *n5* = 0.0001.

**Figure 21:**
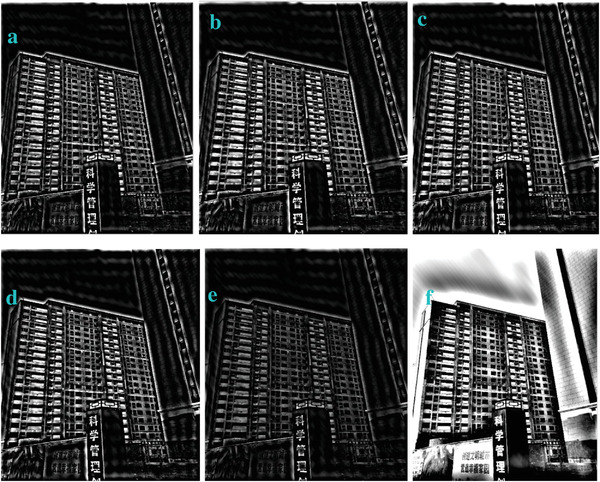
The change of *n5* value for processing the Building image: a) *n5* = 7; b) *n5* = 17; c) *n5* = 26; d) *n5* = 1000; e) *n5* = 0.1; f) *n5* = 0.0001.

Equations (2)–(5) depict the expression of the “cosh‐acosh” filter. We modified the parameter n5 in Equation (3) and got the image effect as shown in **Figures** **17**, **18**, **19**, **20**, **21**. They processed a morphology that is similar to the images made by printmaking. Comparing to those images processed by the “exp” filter, they preserve more details of the original figures.

#### 3.1.3 The “sech‐asech” Filter

**Figure 22:**
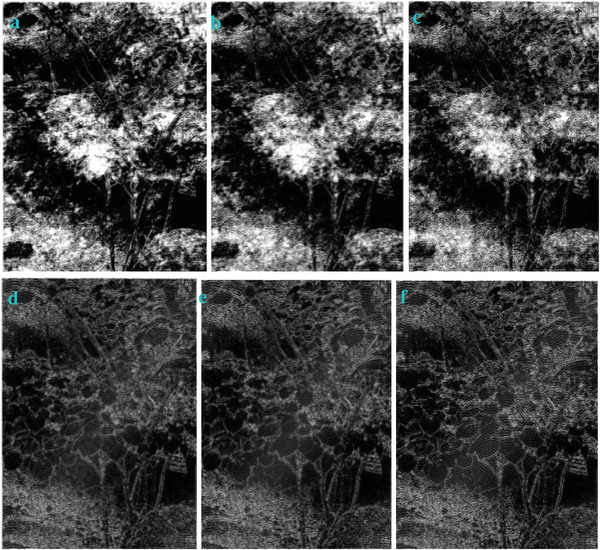
a) *nn* = 24; b) *nn* = 51; c) *nn* = 128; d) *nn* = 128700; e) *nn* = 12870000; f) *nn* = 800000007.

**Figure 23:**
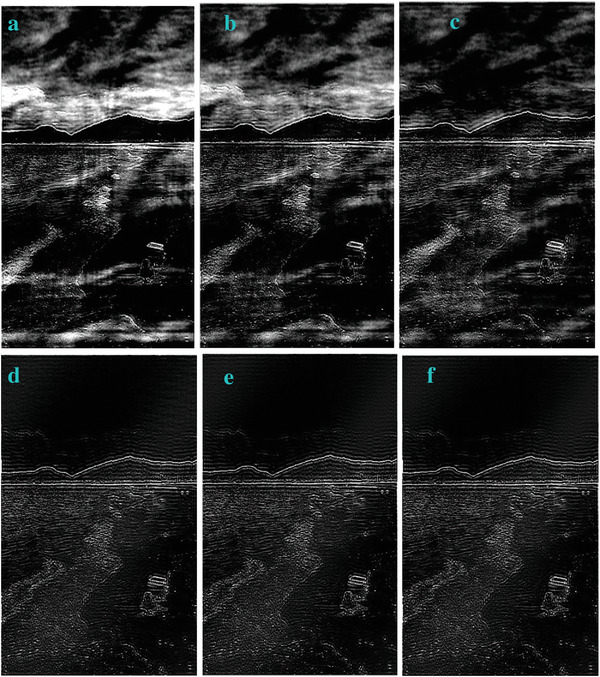
a) *nn* = 24; b) *nn* = 51; c) *nn* = 128; d) *nn* = 128700; e) *nn* = 12870000; f) *nn* = 800000007.

**Figure 24:**
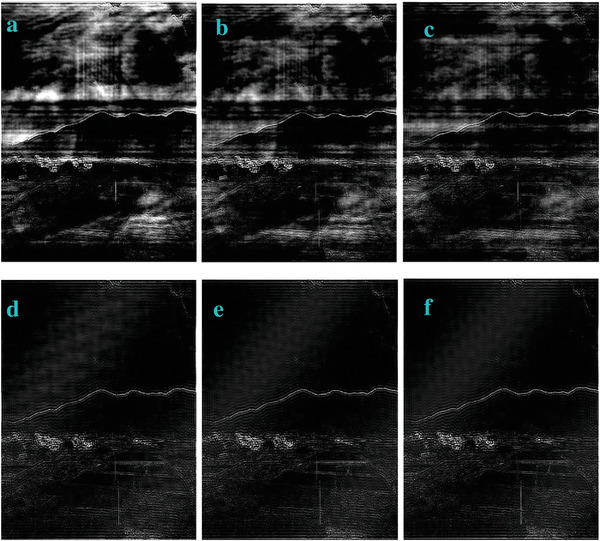
a) *nn* = 24; b) *nn* = 51; c) *nn* = 128; d) *nn* = 128700; e) *nn* = 12870000; f) *nn* = 800000007.

**Figure 25:**
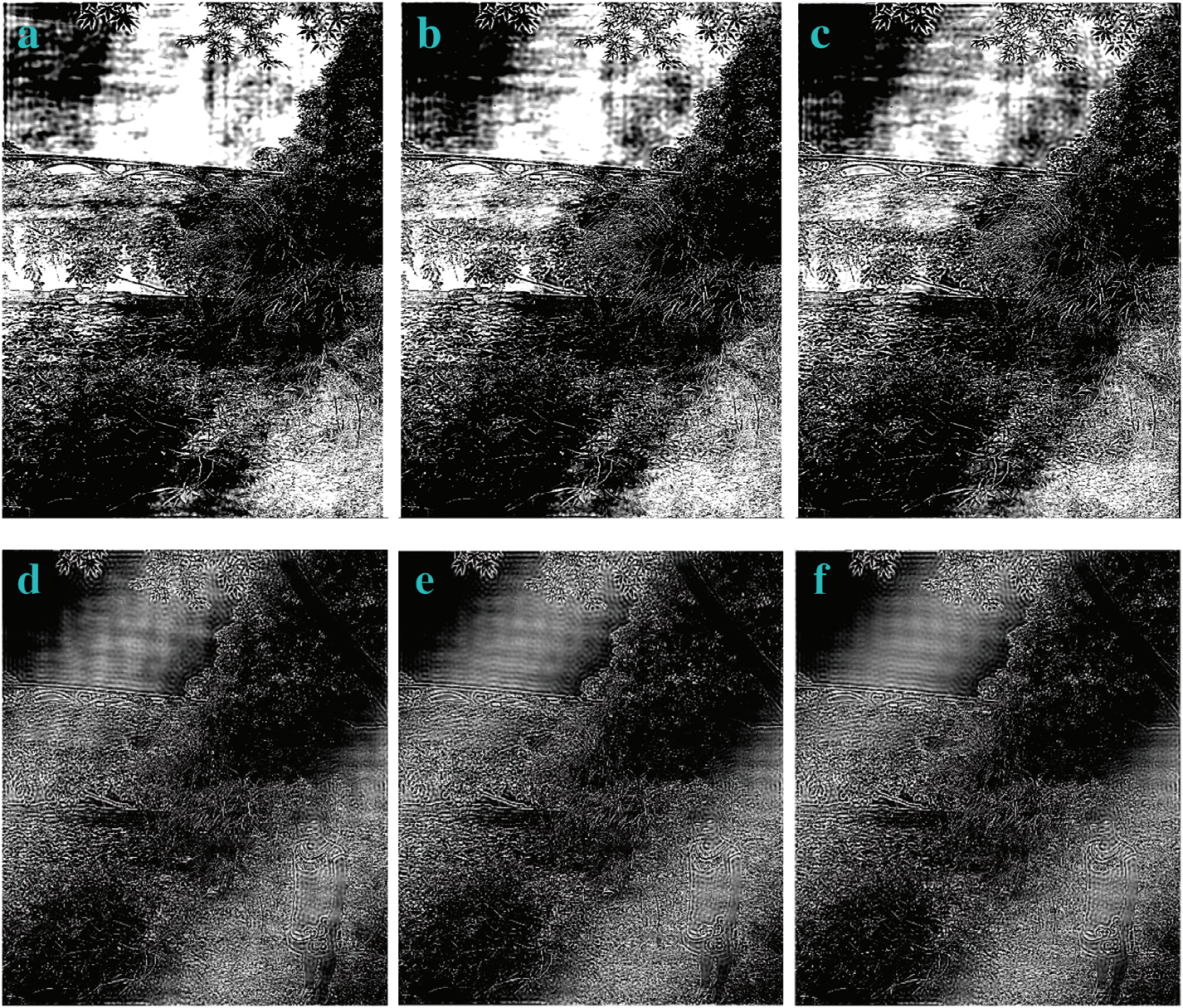
a) *nn* = 24; b) *nn* = 51; c) *nn* = 128; d) *nn* = 128700; e) *nn* = 12870000; f) *nn* = 800000007.

**Figure 26:**
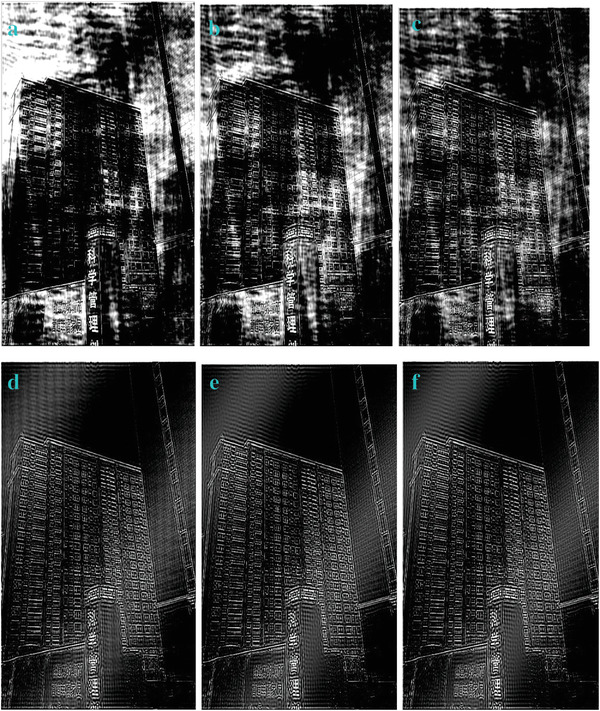
a) *nn* = 24; b) *nn* = 51; c) *nn* = 128; d) *nn* = 128700; e) *nn* = 12870000; f) *nn* = 800000007.

We modify the parameter of nn to get the effect of image edge detection (**Figures** **22**, **23**, **24**, **25**, **26**). When the value of nn is enhanced, only parts of original morphology are shown and the brightness decreases. Some corrugated features are shown in the images.

#### 3.1.4 The “sinh‐asinh‐r” Filter

**Figure 27:**
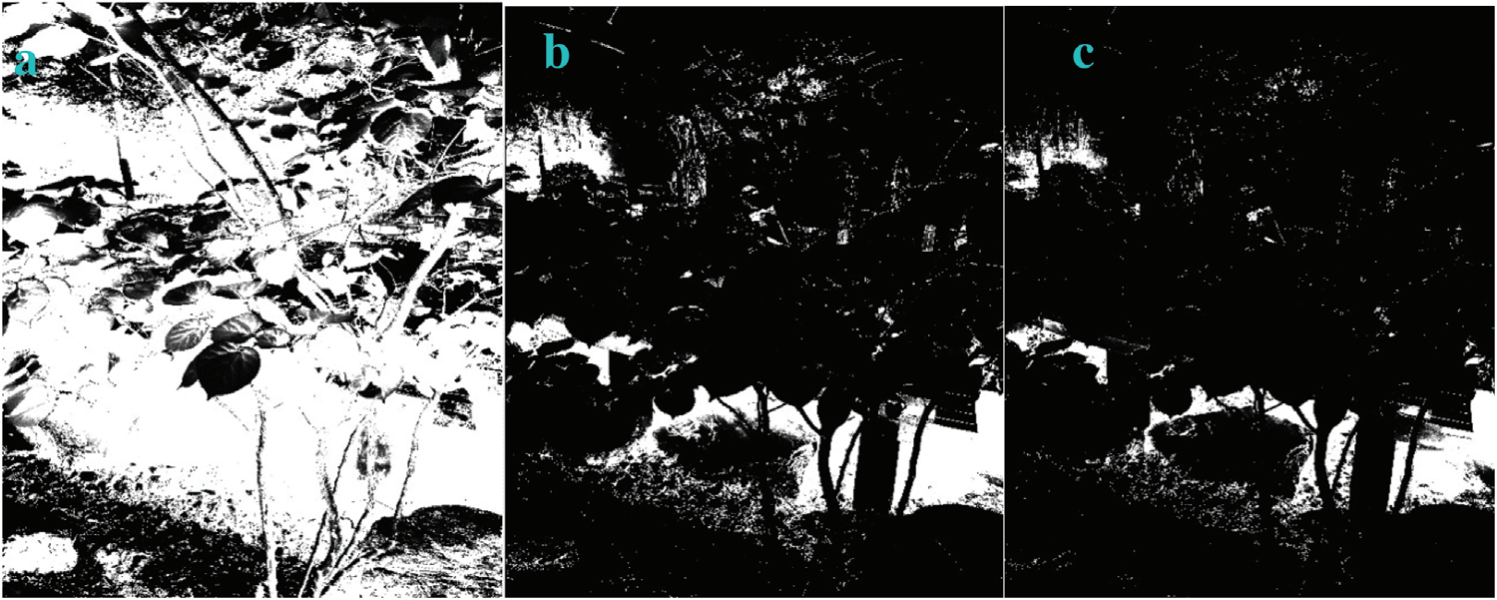
a) *a_3_* = 2; b) *a_3_* = 4; c) *a_3_* = 5.

**Figure 28:**
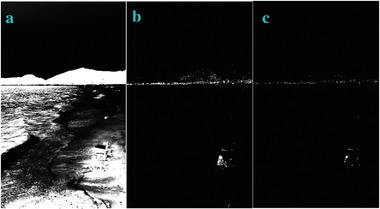
a) *a_3_* = 2; b) *a_3_* = 4; c) *a_3_* = 5.

**Figure 29:**
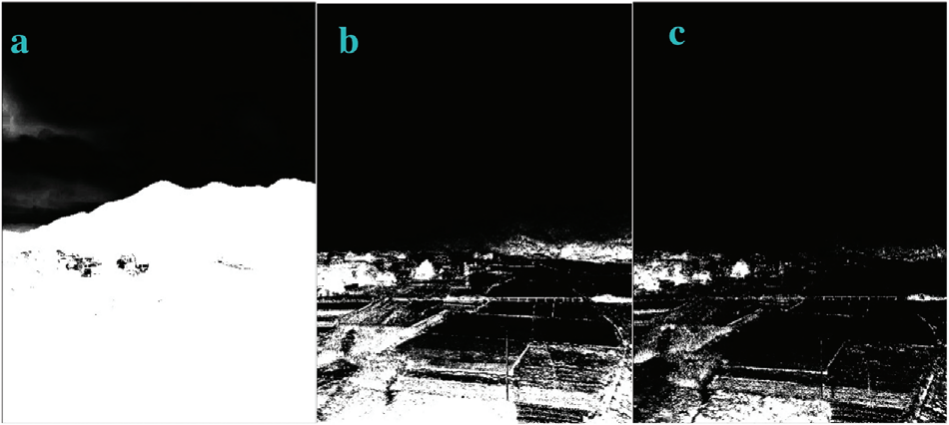
a) *a_3_* = 2; b) *a_3_* = 4; c) *a_3_* = 5.

**Figure 30:**
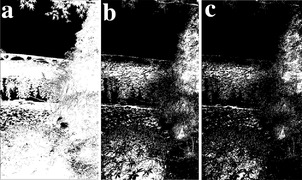
a) *a_3_* = 2; b) *a_3_* = 4; c) *a_3_* = 5.

**Figure 31:**
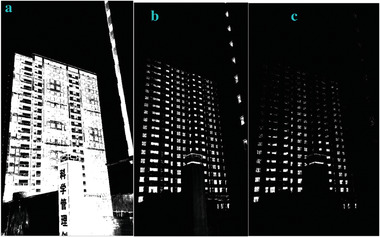
a) *a_3_* = 2; b) *a_3_* = 4; c) *a_3_* = 5.

We modified the parameter of *a_3_* in Equation (10) and got the marginal features (**Figures** **27**, **28**, **29**, **30**, **31**). It should be noted that only *a_3_* is found to be effective for bringing change in the processing. The changing of other parameters showed little variation of the image profile. The increase in *a_3_* will lead to the decrease in image features.

### 3.2 Processing the Images when Gaussian Noises with a High Density of 0.9 are Applied

**Figure 32:**
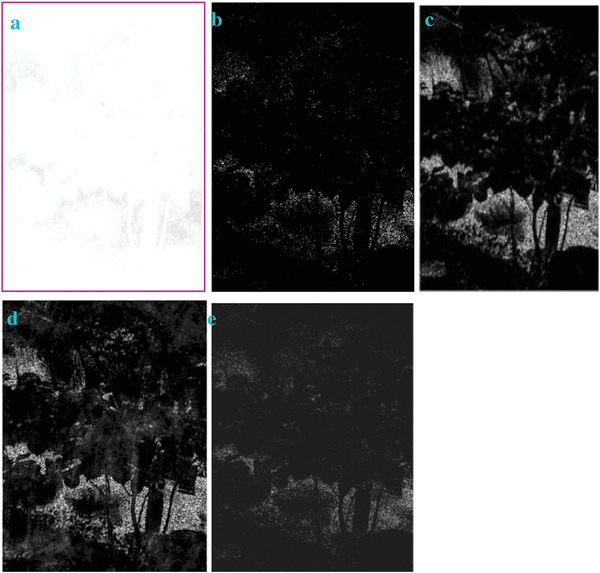
a) The noisy Tree image which is made via adding Gaussian noise to the Tree image. Its noise density is 0.9. Since it contains a lot of noise points, it is hard to be identified. It was processed by several filters: b) the “ exp” filter; c) the “ cosh‐acosh “ filter; d) the “ sech‐asech “ filter; e) the “ sinh‐asinh‐r” filter.

**Figure 33:**
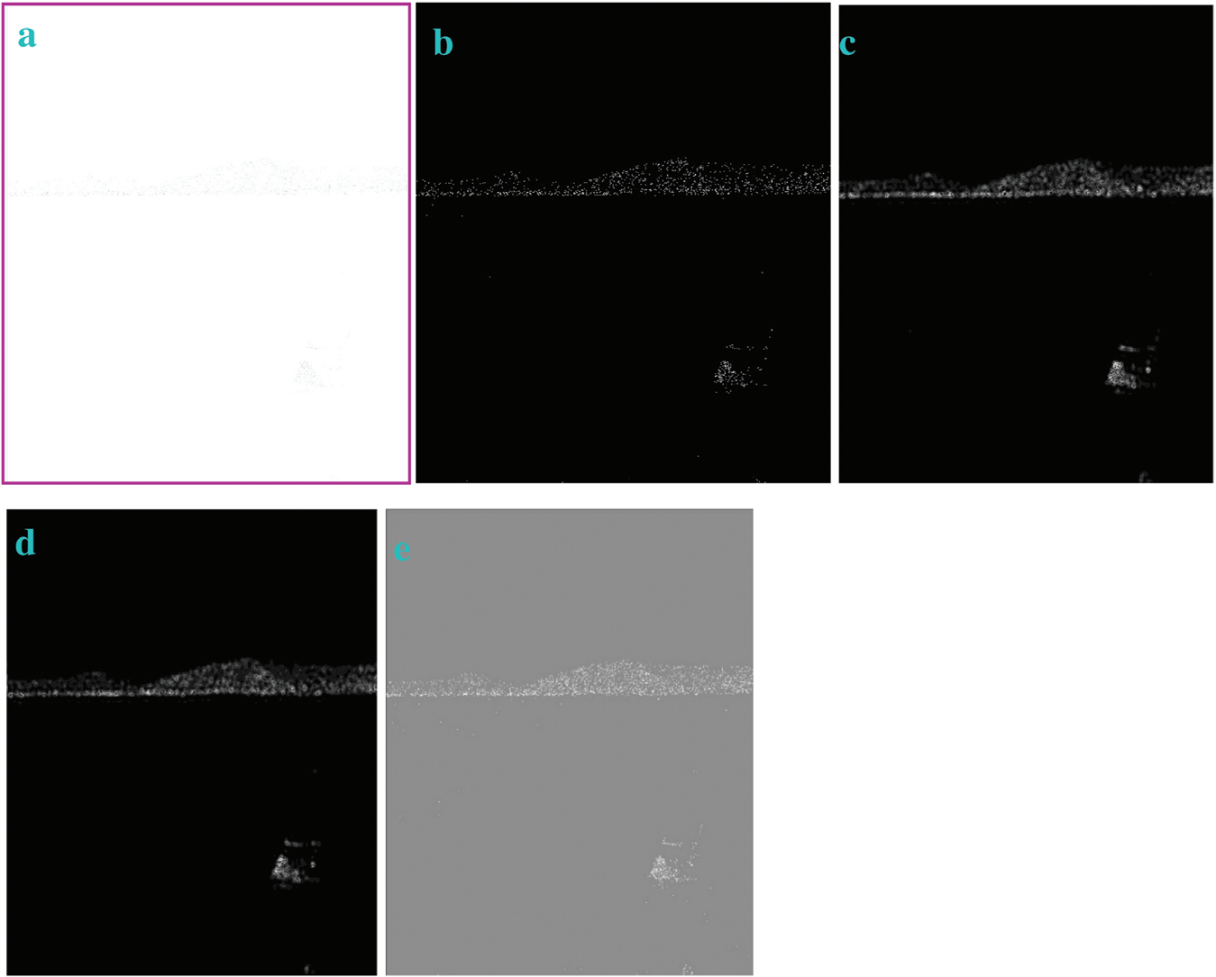
a) The noisy Sea image which is made via adding Gaussian noise to the Sea image. Its noise density is 0.9. It is handled by several filters: b) the “exp” filter; c) the “cosh‐acosh“ filter; d) the “sech‐asech“ filter; e) the “sinh‐asinh‐r” filter.

**Figure 34:**
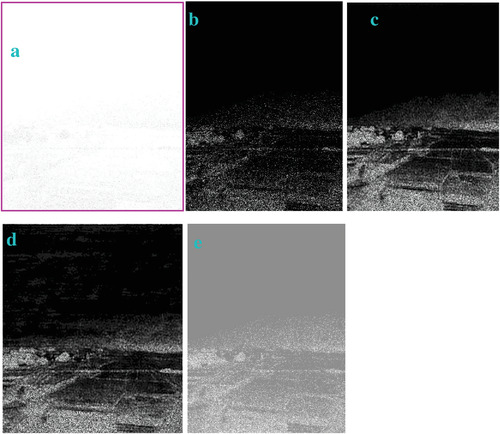
a) The noisy Field image which is made via adding Gaussian noise to the Field image. Its noise density is 0.9. It is processed by several filters: b) the “exp” filter; c) the “cosh‐acosh “ filter; d) the “sech‐asech“ filter; e) the “sinh‐asinh‐r” filter.

**Figure 35:**
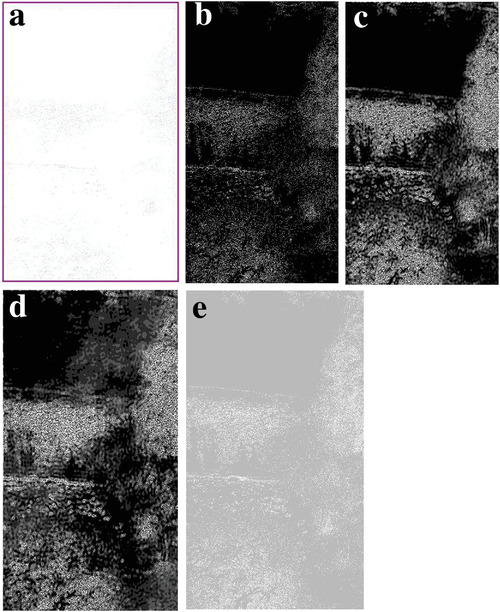
a) The noisy Lakeside image which is made via adding Gaussian noise to the Lakeside image. Its noise density is 0.9. It is processed by several filters: b) the “exp” filter; c) the “cosh‐acosh “ filter; d) the “sech‐asech“ filter; e) the “sinh‐asinh‐r” filter.

**Figure 36:**
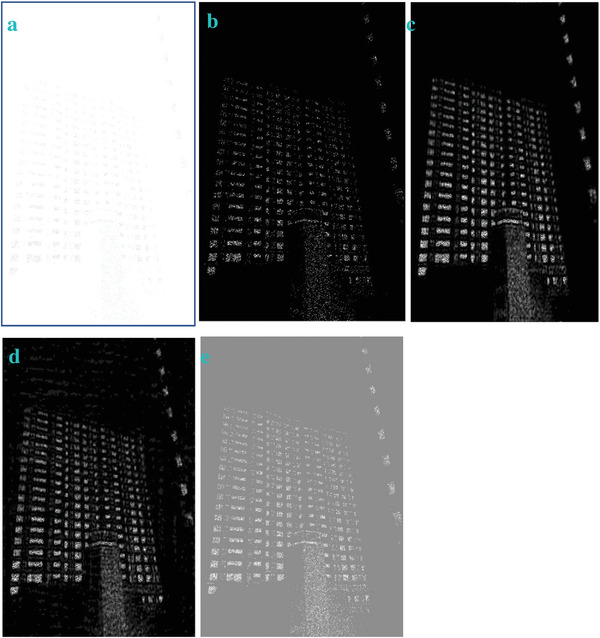
a) The noisy Building image which is made via adding Gaussian noise to the Building image. Its noise density is 0.9. Several filters were used to process this image: b) the “exp” filter; c) the “cosh‐acosh“ filter; d) the “sech‐asech“ filter; e) the “sinh‐asinh‐r” filter.

In most of the real‐life applications, we always have to deal with the images associated with very high noise density. In order to verify that whether these filters shall be useful in processing noisy image, we added Gaussian noise with a very high density of 0.9 to the images (**Figures** **32**a, **33**a, **34**a, **35**a, and **36**a). It can be seen that “white” spots are presented everywhere in the image due to the adding of the Gaussian noise. It gets difficult for people to identify the detailed feature or morphology. Then, we used the “exp” filter, the “cosh‐acosh” filter, the “sech‐asech” filter, and the “sech‐asech‐r” filter to process Figures 32, 33, 34, 35, and 36a. It can be seen from Figures 32b–e, 33b–e, 34b–e, 35b–e, and 36b–e that these filters are very useful for image enhancement. The edge can be extracted after we used these filters no matter the very high noise is presented in the original images.

### 3.3 Comparing to the Traditional Operators

**Figure 37:**
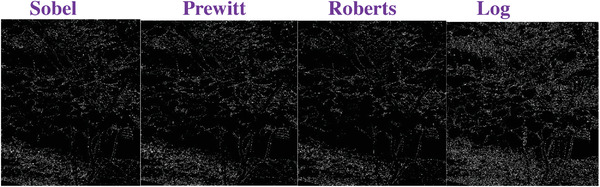
The Tree image processed by the operators of Sobel, Prewitt, Roberts and Log.

**Figure 38:**
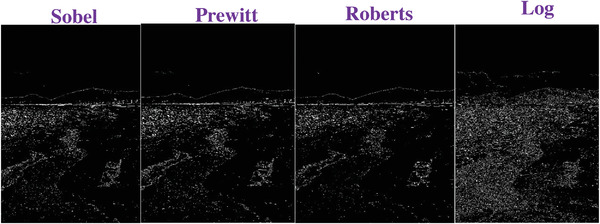
The Sea image processed by the operators of Sobel, Prewitt, Roberts and Log.

**Figure 39:**
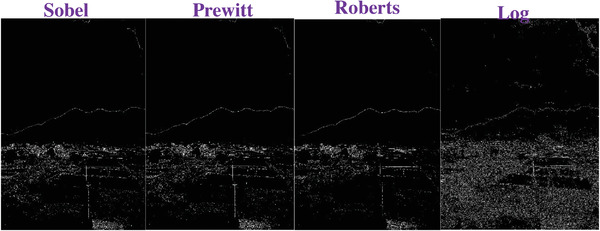
The Field image processed by the operators of Sobel, Prewitt, Roberts and Log.

**Figure 40:**
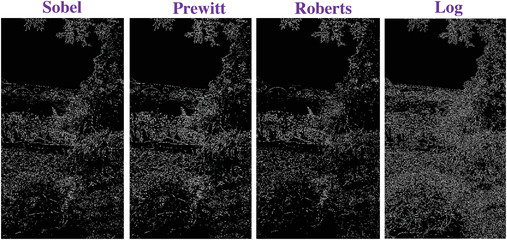
The Lakeside image processed by the operators of Sobel, Prewitt, Roberts and Log.

**Figure 41:**
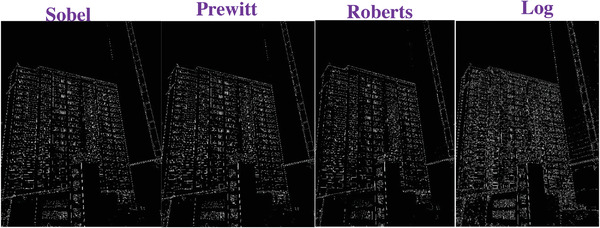
The Building image processed by the operators of Sobel, Prewitt, Roberts and Log.

**Figure 42:**
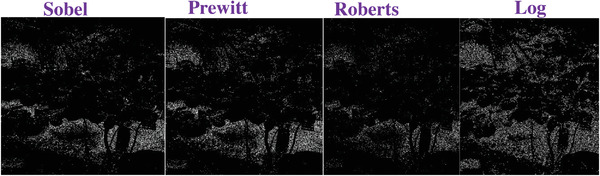
The noisy Tree‐ image processed by the operators of Sobel, Prewitt, Roberts and Log.

**Figure 43:**
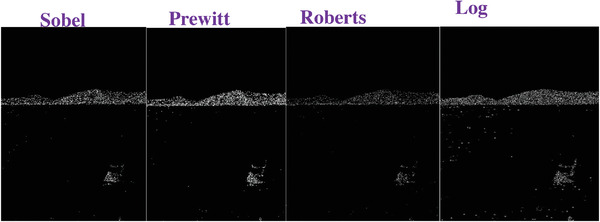
The noisy Sea‐ image processed by the operators of Sobel, Prewitt, Roberts and Log.

**Figure 44:**
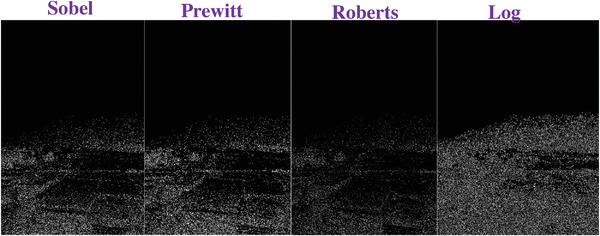
The noisy Field‐ image processed by the operators of Sobel, Prewitt, Roberts and Log.

**Figure 45:**
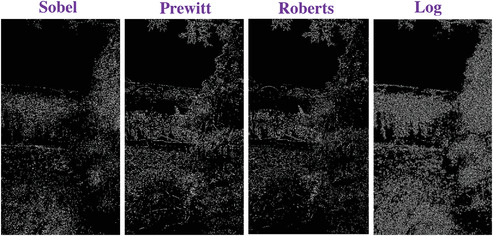
The noisy Lakeside‐image processed by the operators of Sobel, Prewitt, Roberts and Log.

**Figure 46:**
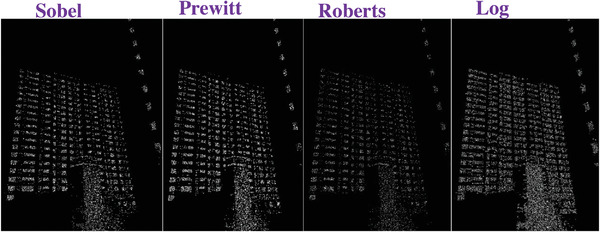
The noisy Building‐image processed by the operators of Sobel, Prewitt, Roberts and Log.

To evaluate the effectiveness of our approach, we compare our designed filters with some traditional operators, including Sobel, Prewitt, Roberts and Log. We used Figures 32, 33, 34, 35, and 36a for processing, which contain a Gaussian noise with the density of 0.9. As shown in **Figures** **37**, **38**, **39**, **40**, **41**, we can extract the edge using these operators. In **Figures** **42**, **43**, **44**, **45**, **46**, we first added Gaussian noise with a density of 0.9 and then performed these operators. Although these operators are useful for getting the edge of the images, some critical features are missing due to the existence of the Gaussian noise. For example, it is pretty hard to identify the marginal details of the branches and leaves of the trees in Figure 45.

### 3.4 Comparing to the Filters Based on the Watershed Algorithm

**Figure 47:**
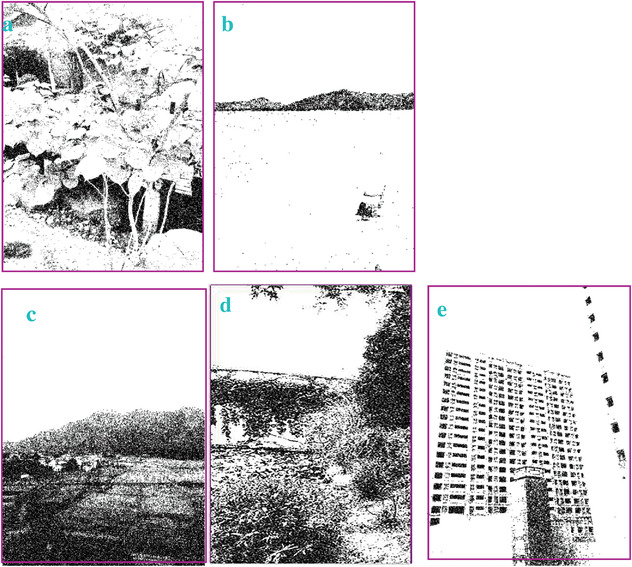
A gaussian noise with noise density of 0.9 was added to the images. The images were processed by the filters based on the watershed algorithm: a) Tree; b) Sea; c) Field; d) Lakeside; e) Building.

We constructed one type of filters based on the watershed algorithm.^[^ ^32^ ^]^ We added a Gaussian noise with noise density of 0.9 to the images of Tree, Sea, Field, Lakeside, and Building. The noisy images can be found in Figure 32, 33, 34, 35, and 36a. Due to the existence of the very high density of the noise, the images of Figure 32, 33, 34, 35, and 36a become white and blur. We used the filters based on the watershed algorithm to process these images. The resulting images can be found in **Figure** **47**a–e. It can be seen that the profiles are successfully extracted from the background of the Gaussian noise. The only problem is that they make the images very bright and keep some noise left in the images.

### 3.5 Comparing to the Filters Based on Gabor Wavelets

**Figure 48:**
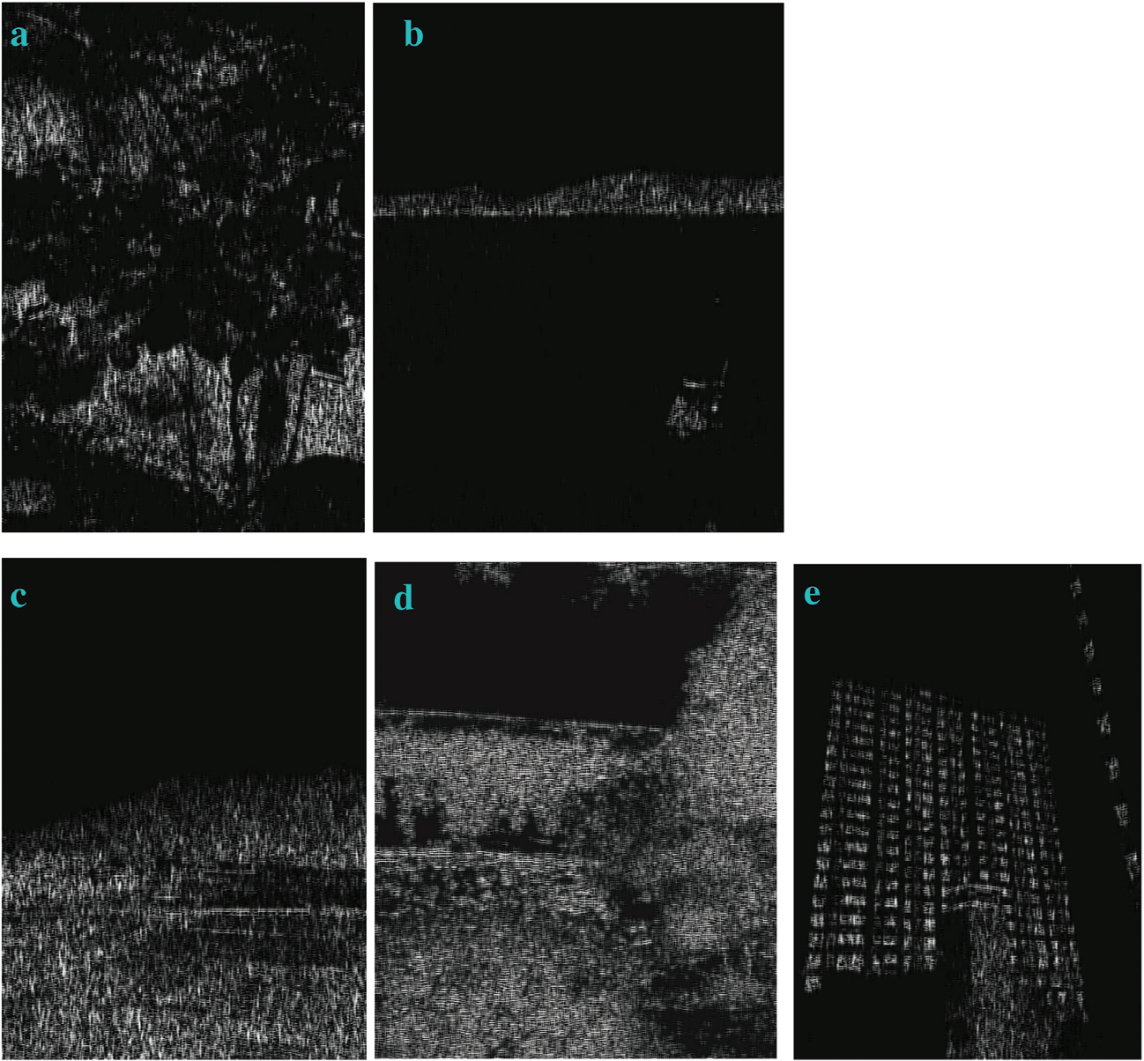
A gaussian noise with noise density of 0.9 was added to the images. The images were processed by the filters based on Gabor wavelets: a) Tree; b) Sea; c) Field; d) Lakeside; e) Building.

For those images of Figures 32, 33, 34, 35, and 36a, we processed them using filters based on Gabor wavelets.^[^ ^33^ ^]^ It can be seen from **Figure** **48** that images profiles were extracted. They are very similar to the images processed by the “exp” filter (Figures 32, 33, 34, 35, and 36b).

### 3.6 Comparing to Matched Filters

**Figure 49:**
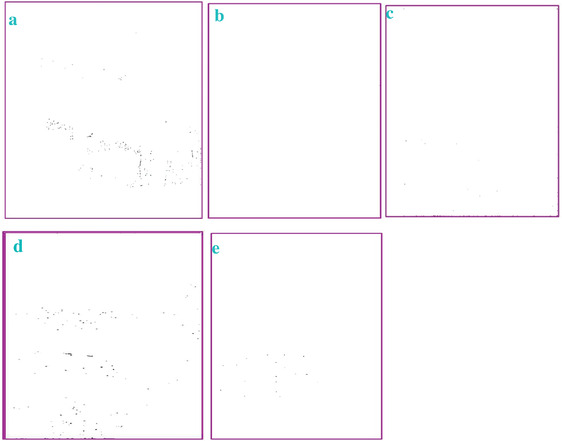
A gaussian noise with noise density of 0.9 was added to the images. The images were processed by the matched filters: a) Tree; b) Sea; c) Field; d) Lakeside; e) Building.

**Figure 50:**
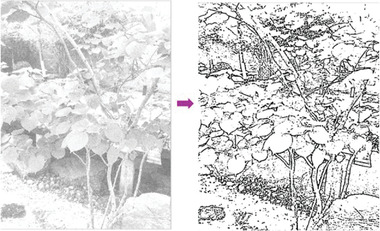
A Gaussian noise with noise density of 0.5 was added to the image Tree (left). It was processed by the matched filter (right).

**Figure 51:**
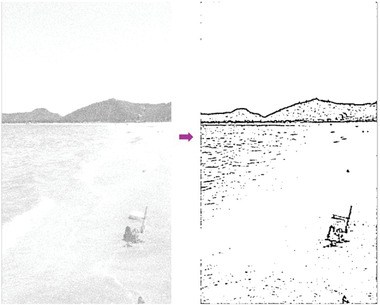
A Gaussian noise with noise density of 0.5 was added to the image Sea (left). It was processed by the matched filter (right).

**Figure 52:**
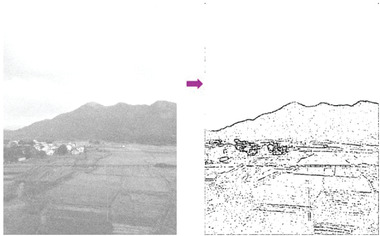
A Gaussian noise with noise density of 0.5 was added to the image Sea (left). It was processed by the matched filter (right).

**Figure 53:**
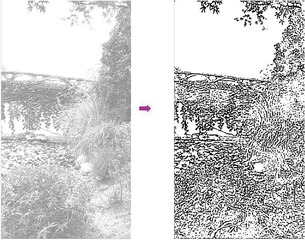
A Gaussian noise with noise density of 0.5 was added to the image Lakeside (left). It was processed by the matched filter (right).

**Figure 54:**
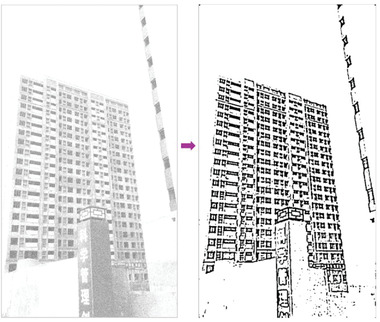
A Gaussian noise with noise density of 0.5 was added to the image Building (left). It was processed by the matched filter (right).

We constructed the matched filters^[^ ^34^ ^]^ to process those images of Figures 32, 33, 34, 35, and 36a. It turned out that the matched filters cannot be effective in such images containing high level noise. The filtered images are completely bright and only several spots can be seen in the images (**Figure** **49**a–e). If we decrease the noise density to the level of 0.5, we are able to extract the profile using the matched filters (**Figures** **50**, **51**, **52**, **53**, **54**).

## 4 Discussion

### 4.1 Advances of Our Technology and Method

On the road of exploring image edge detection, many excellent edge detection operators have been discovered. These classical edge detection operators have been modified to get new methods for image edge detection. These filters are all classical and incredible. Our work provides a supplement to these existing solutions. One advantage of our works is that our solution is very flexible since it is based on the designing of the special functions. The parameters in the functions can be modified in order to get specific processing.

Moreover, our work has provided a basic framework. With this framework, we can integrate more functions into it and design a new image filter with much more functions. This may lead to a wider range of applications. Our work is very original. It is rare to find out that other scholars have used similar special function to form a filter for image edge detection.

However, due to the complexity of the image, the effect of filter processing may be different for different images. For example, when the image contains a lot of noise or the image has low contrast and brightness, the processing effect may be poor or need to be adjusted in terms of the brightness or contrast.

Beyond that, the next thing we have to do is to make our filters much more useful for the users. Our future work would be designing a graphical user interface based on our filters.

### 4.2 Possible Mechanism of the Effectiveness of These Filters

It can be seen that our designed filters are capable of getting image edge profile. When high noise was applied to the image, they can be used to get rid of the noise and achieve clear edge profile. This may be due to the robustness of the special functions we used, such as the hyperbolic functions. The exact origin of the effectiveness of these filters for edge detection would be our future endeavor.

### 4.3 Potential Application in Near Infrared Imaging

**Figure 55:**
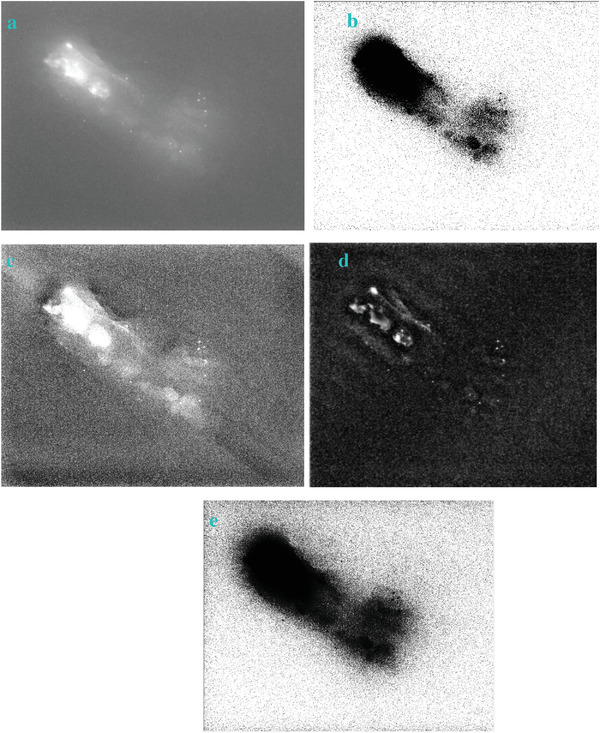
a) The image from near infrared imaging; The image processed by several filter: b) the “exp” filter; c) the “cosh‐acosh” filter; d) the “sech‐asech” filter; e) the “sinh‐asinh‐r” filter. The scale bar is 10 mm.

We processed an image acquired from near infrared imaging using the filters of “exp”, “cosh‐acosh”, “sech‐asech”, and “sinh‐asinh‐r” separately. The detailed technique and instrumentation of the near infrared imaging can be found elsewhere.^[^ ^35^ ^]^ **Figure** **55**a is a centrifuge tube buried below pig tissue. This tube is filled with dye solution. The dye molecules can emit near infrared fluorescence, which can be detected by the near infrared camera. The infrared fluorescence holding long optical wavelength can penetrate the thick pig tissue. Therefore, we are able to see the shape of the tube even if it is buried below the tissue.

As we can see from the Figure 55b–e, the proposed filters are useful to extract the profile of the tube. The shape of the tube is enhanced. Its edge is extracted. The “exp” filter and the “sinh‐asinh‐r” filter look very useful for edge extraction. It has shown the clear shape and edge of the tube. The image processed by the “cosh‐acosh” filter has exhibited sharp edge. Relatively, the image processed by the “sech‐asech” filter has shown very dark feature, which showed some limitation of this filter.

In the field of video monitoring, the technology of near infrared imaging is widely used. One major advance is that they show the merit of the vast accuracy of the recognition as well as the long imaging depth.^[^ ^36^ ^]^ They are generally combined with visual light imaging to get the precise detection. Our designed functions have shown flexible structures, which may be modified to be used in this field. Moreover, we did not consider the calculation efficiency of these functions in current design. In our future work, we have to continue to make them adaptable for the increasing demand on giant computer systems,^[^ ^36^ ^]^ internet of things,^[^ ^37^ ^]^ and wireless sensor networks.^[^ ^38^, ^39^, ^40^ ^]^

Modern military systems like scout planes and drones used cameras to perform tracking and analysis. Low‐quality images can be taken when poor outdoor conditions compromise their performance. It is suggested that deep learning, which is essentially a neural network with layers, is one of the effective and emerging techniques for enhancement of those images.^[^ ^41^, ^42^, ^43^, ^44^ ^]^ It is interesting to find out that it is strongly depended on the training datasets. Therefore, it can be used to process various low‐quality images generated by the sophisticated environment that is associated with low illumination levels, strong color deviations, complex artifacts, high‐level noise, etc. This advantage is something that our proposed framework cannot compare, which can only be useful for processing the low‐quality images associated with the noise. One of our future work direction would be focused on the modification of our method in order to make them useful for processing various low‐quality images.

**Figure 56:**
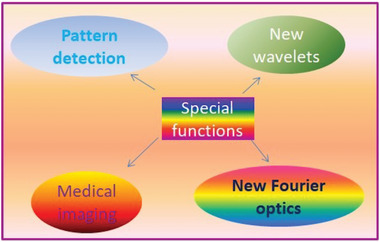
Proposed future applications of special functions.

**Figure** **56** illustrates the proposed future applications of the special functions. Indeed, the Fourier optics is generally dealing with Fourier transform using the hardware of lens and optical parts. Our proposed special functions are actually a special form of Fourier transform. Its experimental implementation can be complex, which can bring new Fourier optics. Moreover, the development of novel wavelets are always in high demand for technological applications.^[^ ^45^, ^46^, ^47^, ^48^, ^49^, ^50^, ^51^, ^52^, ^53^ ^]^ Our designed special function can be combined with time depended parameter that makes them very useful for constructing a new form of the wavelets.

## 5 Conclusion

Image enhancement has always been an important part of computer image vision. Although there are many methods developed today, the study of special functions based filters is still an area that has not yet been stepped in. This article proposes an effective and flexible framework for the image enhancement via using four kinds of filters. First, this study has presented a filter based on the exponential function. It is found that the image feature is related with the value of progression. When the value of the progression is even, the images show edge feature. When the value of the progression is odd, the images show sharp contrast. Second, this study has used hyperbolic cosine and its inverse function to build a filter, where a printmaking effect can be shown. Third, we constructed a filter based on a hyperbolic secant function and its inverse. The variation of the progression value will lead to the effect of image edge detection. When the progression value is increasing, marginal morphology is shown and the brightness is suppressed. It can be seen that some ripple features are existing in the images. Fourth, we built a filter based on a hyperbolic sine function and its inverse, where marginal features can be extracted.

Moreover, they have shown a good suppression effect on the Gaussian noise. The marginal features can be extracted even when a high noise density of 0.9 is presented in the original images. They can also be useful for highlighting the images acquired from near infrared imaging.

Potential applications of our methods in the pattern monitoring and the medical imaging can be expected. These special functions are proposed to be useful in the field of creating the new Fourier optics and novel forms of the wavelets functions.

## Conflict of Interest

The authors declare no conflict of interest.

## Author Contributions

R.Y., L.C., Z.L., and Y.W. performed conceptualization and methodology. L.Z. and Z.L. administered the project. R.Y., Y.L., and Y.W. wrote and prepared original draft. Y. L. and Y.W. performed writing review and editing. All authors have read and agreed to the published version of the manuscript.

## Data Availability

Research data are not shared.
